# Nitrogen-incorporation activates NiFeO_x_ catalysts for efficiently boosting oxygen evolution activity and stability of BiVO_4_ photoanodes

**DOI:** 10.1038/s41467-021-27299-0

**Published:** 2021-11-29

**Authors:** Beibei Zhang, Shiqiang Yu, Ying Dai, Xiaojuan Huang, Lingjun Chou, Gongxuan Lu, Guojun Dong, Yingpu Bi

**Affiliations:** 1 State Key Laboratory for Oxo Synthesis & Selective Oxidation, National Engineering Research Center for Fine Petrochemical Intermediates, Lanzhou Institute of Chemical Physics, CAS, 730000 Lanzhou, P. R. China; 2 University of Chinese Academy of Sciences, 100049 Beijing, P. R. China; 3 School of Physics, Shandong University, 250100 Jinan, P. R. China; 4 Dalian National Laboratory for Clean Energy, CAS, 116023 Dalian, P. R. China

**Keywords:** Materials for energy and catalysis, Nanoscale materials, Photochemistry

## Abstract

Developing low-cost and highly efficient catalysts toward the efficient oxygen evolution reaction (OER) is highly desirable for photoelectrochemical (PEC) water splitting. Herein, we demonstrated that N-incorporation could efficiently activate NiFeO_x_ catalysts for significantly enhancing the oxygen evolution activity and stability of BiVO_4_ photoanodes, and the photocurrent density has been achieved up to 6.4 mA cm^−2^ at 1.23 V (vs. reversible hydrogen electrode (RHE), AM 1.5 G). Systematic studies indicate that the partial substitution of O sites in NiFeO_x_ catalysts by low electronegative N atoms enriched the electron densities in both Fe and Ni sites. The electron-enriched Ni sites conversely donated electrons to V sites of BiVO_4_ for restraining V^5+^ dissolution and improving the PEC stability, while the enhanced hole-attracting ability of Fe sites significantly promotes the oxygen-evolution activity. This work provides a promising strategy for optimizing OER catalysts to construct highly efficient and stable PEC water splitting devices.

## Abstract (web summary)

While solar-to-fuel conversion offers a renewable energy production strategy, photoelectrochemical water splitting requires further optimization. Here, authors examined nitrogen-doped NiFeO_x_ catalysts to improve the oxygen evolution activity and stability of BiVO_4_ photoanodes.

## Introduction

Photoelectrochemical (PEC) water splitting has been considered as a promising strategy for converting solar light into hydrogen energy^1–3^. To achieve its practical applications, the design and fabrication of semiconductor photoanodes with sufficient light absorption, effective charge separation, and high surface reactivity are essentially required^4,5^. Among various candidates, bismuth vanadate (BiVO_4_) has been attracted particular attentions owing to its appropriate bandgap (2.4 eV) and suitable band-edge positions^6–10^. However, suffering from the high charge-recombination and sluggish oxygen evolution reaction (OER) kinetics, most of reported photocurrent densities of BiVO_4_ photoanodes are far below the theoretical expectation (7.5 mA cm^−2^, AM 1.5 G illumination, 100 mW cm^−2^)^11–13^. During past decades, diverse strategies have been developed to improve the PEC activities of BiVO_4_ photoanodes, including elemental doping^14–16^, facet tailoring^17–19^, and hetero-junction^20–24^, etc. Although the PEC performances have been increased to a certain extent owing to the improved carrier mobility as well as electrical conductivity, the intrinsically poor surface reactivity still seriously restricts the PEC conversion efficiency.

Recently, BiVO_4_ photoanodes decorated with various transition-metal catalysts have been extensively reported for remarkably promoting the OER activities^25–29^. Specifically, they could efficiently extract photo-generated holes, minimize over potential, and provide active sites, which are all beneficial to accelerate the PEC water oxidation kinetics. Among various OER catalysts, the VIII metal (Fe, Co, Ni) oxides or (oxy)hydroxides, especially for NiFe-based materials, have attracted particular interests in recent years^30–34^. For example, Domen et al^35^. deposited NiFe bimetallic catalyst on BiVO_4_ photoanodes for improving the PEC activities up to 4.2 mA cm^−2^ at 1.23 V_RHE_. Zhang and co-workers reported that BiVO_4_ photoanodes modified with NiFe complexes exhibited an excellent photocurrent of 5.10 mA cm^−2^ ^31^. Pihosh et al.^36^ fabricated a WO_3_/BiVO_4_/CoPi core-shell nanostructured photoanode that achieves near 90% of the theoretical water splitting photocurrent. On this basis, Kosar et al.^37^ acquired a highly efficient solar-to-hydrogen conversion efficiency of 7.7% by photovoltaic cell and WO_3_/BiVO_4_/CoPi core-shell nanorods PEC cell tandem. Despite the crucial roles of OER catalysts for enhancing PEC behaviors have been well established, much less attentions focused on optimizing their electronic structures to further boost the PEC conversion efficiency, especially for bimetallic catalysts.

Herein, we reported the incorporation of non-metallic nitrogen-atom into NiFeO_x_ catalysts to rationally tailor the electronic structure, which remarkably promoted the photocurrent density of BiVO_4_ photoanodes up to 6.4 mA cm^−2^ at 1.23 V_RHE_ under AM 1.5 G (100 mW cm^−2^) with an excellent durability. The outstanding PEC performances should be attributed to the electronic reconstruction in both NiFeO_x_ and BiVO_4_, resulting from the partial substitution of O sites by low electronegativity N atoms. Specifically, the weak electron-attracting capacity of N atoms led to the electron enrichments on both Fe and Ni sites. Subsequently, the electron injection from Ni atoms to lattice V sites of BiVO_4_ was favorable for improving the oxygen-evolution stability, while the Fe sites could effectively attract holes for promoting the PEC activity. This work firstly demonstrates the rational regulation of electronic structures in OER catalysts as well as fundamental understanding of their intrinsic roles in PEC oxygen evolution reaction.

## Results

### Morphology and structure characterizations

**Fig. 1:**
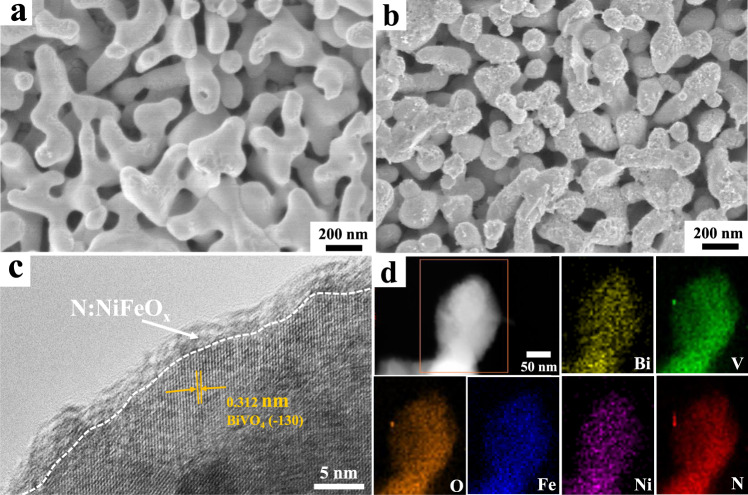
Morphology and structure characterization of the synthetic photoanodes. SEM images of pristine BiVO_4_ (**a**) and BiVO_4_/N:NiFeO_x_ (**b**) photoanodes; HR-TEM image (**c**) and TEM-EDS element mapping analysis (**d**) of BiVO_4_/N:NiFeO_x_ photoanodes.

The nanoporous BiVO_4_ photoanodes supported on F-doped SnO_2_ (FTO) glass substrates were fabricated by an electrochemical deposition associated with calcination treatment^10^. Figure 1a shows the scanning electron microscopy (SEM) images of the obtained BiVO_4_ photoanodes, clearly revealing their unique worm-like porous structure with an average diameter of 200–300 nm. Additionally, the high-resolution transmission electron microscopy (HR-TEM) image (Supplementary Fig. 1) clearly indicates that these nanocrystals possess a relatively smooth surface and a lattice spacing of 0.311 nm corresponded to (−130) plane of monoclinic BiVO_4_ phase. Interestingly, after the decoration of N:NiFeO_x_ catalysts, the smooth surfaces of pristine BiVO_4_ photoanodes transformed into a rough flocculent-structure (Fig. 1b). The HR-TEM images (Fig. 1c and Supplementary Fig. 2) clearly indicate that an amorphous layer of N:NiFeO_x_ catalysts was uniformly covered on BiVO_4_ surfaces with a thickness of ~4 nm. Moreover, Fig. 1d and Supplementary Fig. 3 show the energy dispersive spectroscopy (EDS) elemental line and mapping images, revealing the uniform distributions of N, Ni and Fe elements on BiVO_4_ crystal surfaces. Besides, the X-ray photoelectron spectroscopy (XPS) result also confirms the successful incorporation of the nitrogen element into the NiFeO_x_ layer (Supplementary Fig. 4). However, compared with pristine BiVO_4_ photoanodes, no evident peak change could be observed in the X-ray diffraction (XRD) patterns after the decoration of N:NiFeO_x_ catalysts (Supplementary Fig. 5), which should be due to their amorphous structure and ultrathin thickness.

### Photoelectrochemical properties

**Fig. 2:**
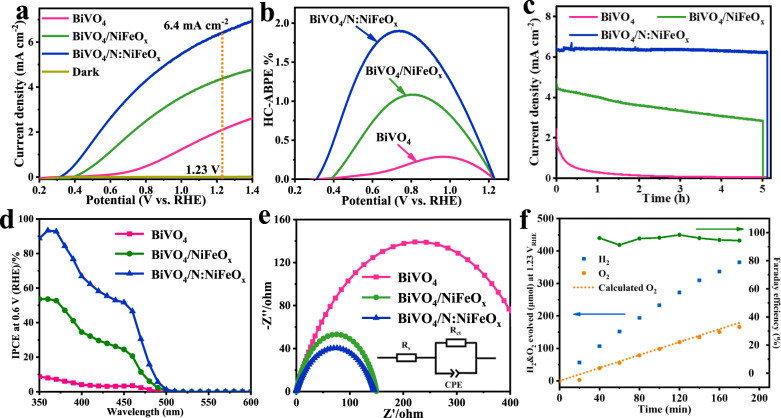
Photoelectrochemical properties. **a** Linear-sweep voltammograms (LSV, with a scan rate of 10 mV s^−1^), **b** half-cell ABPE (HC-ABPE) results, **c** I–t stability tests measured at 1.23 V vs. RHE, **d** IPCE results at 0.6 V vs. RHE and **e** EIS results at 0.75 V vs. RHE under illumination for BiVO_4_, BiVO_4_/NiFeO_x_, and BiVO_4_/N:NiFeO_x_ photoanodes. **f** H_2_ and O_2_ evolution of BiVO_4_/N:NiFeO_x_ measured at 1.23 V vs. RHE. All the measurements were carried at 0.5 M K_3_BO_3_ (pH = 9.5) electrolyte.

The PEC water splitting performances of N:NiFeO_x_ catalyst decorated BiVO_4_ photoanodes (marked as BiVO_4_/N:NiFeO_x_) were measured in 0.5 M K_3_BO_3_ (pH = 9.5) electrolyte under AM 1.5 G illumination (100 mW cm^−2^). For comparison, the PEC activities of pristine BiVO_4_ as well as NiFeO_x_ decorated BiVO_4_ photoanodes (marked as BiVO_4_/NiFeO_x_) have also been studied. As shown in Fig. 2a and Supplementary Fig. 6, the pristine BiVO_4_ photoanodes exhibit a relatively low photocurrent density (2.1 mA cm^−2^ at 1.23 V_RHE_), suffering from the sluggish oxygen evolution kinetics at anode/electrolyte interfaces. Obviously, the decoration of NiFeO_x_ catalysts on BiVO_4_ photoanodes could effectively enhance the PEC water oxidation activity, and the photocurrent density has been increased up to 4.4 mA cm^−2^ at 1.23 V_RHE_. Amazingly, an outstanding photocurrent density of 6.4 mA cm^−2^ at 1.23 V_RHE_ has been achieved on BiVO_4_/N:NiFeO_x_ photoanodes accompanied by a lower onset potential for OER (Supplementary Fig. 7), clearly indicating that the incorporation of N-atom in NiFeO_x_ catalysts could significantly promote the oxygen evolution activity (Supplementary Fig. 8 and Figs. 9 and 10). Furthermore, their maximum half-cell applied bias photon to current efficiencies (HC-ABPE) have been calculated and shown in Fig. 2b. The HC-ABPE value of BiVO_4_/N:NiFeO_x_ photoanode could be achieved up to 1.9% at 0.73 V_RHE_, which is much higher than that of BiVO_4_/NiFeO_x_ (1.1% at 0.8 V_RHE_) and pristine BiVO_4_ (0.29% at 0.96 V_RHE_), respectively. Except for the high conversion efficiency, the high stability and durability of photoelectrodes are also required for future practical applications. Figure 2c shows the current-time (*i-t*) curves of these photoanodes operated at 1.23 V_RHE_. Note that due to the serious photo-corrosion and V^5+^ dissolution from crystal lattices, the pristine BiVO_4_ exhibited the relatively poor PEC stability and the photocurrent density rapidly decreased^38–40^. Although the loading of NiFeO_x_ catalysts on BiVO_4_ surfaces could improve the PEC stability to a certain extent, the photocurrent density also decreased down to 2.8 mA cm^−2^ after 5 h test. Interestingly, BiVO_4_/N:NiFeO_x_ photoanodes possess the excellent photocurrent stability during the whole test process, indicating the positive effects of N:NiFeO_x_ on restraining V^5+^ dissolution from BiVO_4_ lattices and the obtained photoanodes with excellent structural stability (Supplementary Fig. 11, Figs. 12 and 13, and Table 1). The above results clearly reveal that the incorporation of N atoms in NiFeO_x_ catalysts not only significantly promotes the oxygen evolution activity but also effectively enhances the PEC stability of BiVO_4_ photoanodes.

Furthermore, their incident photon to current conversion efficiencies (IPCEs) were conducted and shown in Fig. 2d (Supplementary Fig. 14). At the wavelength of 360 nm, the IPCE values of BiVO_4_/N:NiFeO_x_ photoanodes could be achieved to 93%, which is much higher than BiVO_4_ (8%) and BiVO_4_/NiFeO_x_ (54%). Figure 2e shows the electrochemical impedance spectroscopy (EIS) for further elucidating the interfacial charge transfer and oxygen evolution kinetic. According to the Nyquist plots and the fitting results (Supplementary Table 2), the calculated resistance values of BiVO_4_/N:NiFeO_x_, BiVO_4_/NiFeO_x_, and BiVO_4_ photoanodes were 139.5, 149.8, and 458.9 Ω, respectively, revealing the preferable capability of N:NiFeO_x_ catalyst for facilitating interface charge transfer. Moreover, the hydrogen and oxygen amounts generated from PEC water splitting over BiVO_4_/N:NiFeO_x_ photoanodes were measured by an online gas chromatography (GC). After 3 h irradiation, the amounts of H_2_ and O_2_ increased linearly up to 365.3 and 165.8 μmol, respectively (Fig. 2f). Additionally, an average Faradaic efficiency of nearly 95% has been obtained on BiVO_4_/N:NiFeO_x_ photoanodes, further confirming its excellent oxygen evolution capability.

### Spectrum and electrochemical analysis

**Fig. 3:**
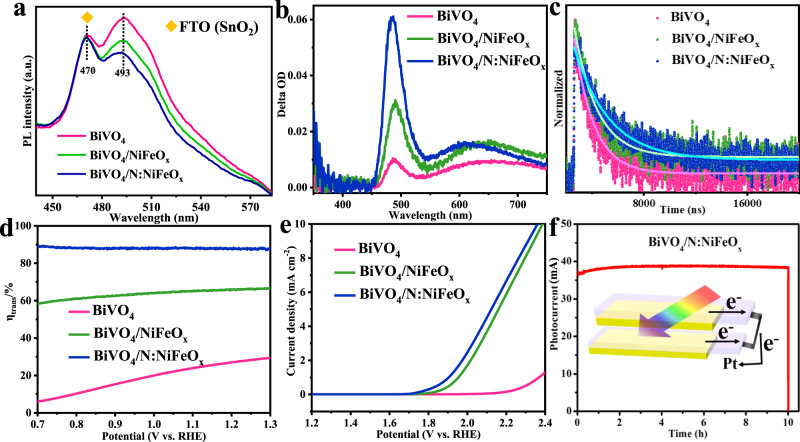
Spectrum and electrochemical OER properties. **a** PL spectra; **b** transient absorption (TA) spectra; **c** the time-resolved TA curves probed at 490 nm; **d** charge transfer efficiencies (η_trans_); and **e** LSV curves in the dark of BiVO_4_, BiVO_4_/NiFeO_x_, and BiVO_4_/N:NiFeO_x_ photoanodes. **f** I–t curves of the scale-up fabricated BiVO_4_/N:NiFeO_x_ photoanodes (2 × 3.5 cm^2^) with parallel at 1.23 V_RHE_.

The photoluminescence spectroscopy (PL) has been measured by a fluorescence spectrophotometer under laser excitation of 355 nm. As shown in Fig. 3a, two PL peaks could be clearly identified. More specifically, the peak at 470 nm is associated with FTO (SnO_2_) substrate (Supplementary Fig. 15). The peak at 493 nm near the absorption band edge of BiVO_4_ (Supplementary Fig. 16) is attributed to radiative recombination of hole in O 2p band and electron in V 3d band, which represents the charge recombination ability^41,42^. Specifically, the pristine BiVO_4_ photoanodes exhibited a very strong PL peak, demonstrating the relatively high electron-hole recombination ratios. However, after the decoration of OER catalysts, the PL peak intensities have been evidently reduced. More specifically, N:NiFeO_x_ catalysts exhibit more efficient capability than NiFeO_x_ for promoting the charge separation of BiVO_4_ photoanodes (Supplementary Fig. 17). Moreover, the time-resolved transient absorption spectra (TR-TAS) have been performed to explore the energy relaxation process and the charge carrier concentrations of the related samples under the excited state^43–45^. In addition, the decay curves were probed at 490 nm, which was attributed to hole dynamics^43^. As shown in Fig. 3b, c (Supplementary Table 3), the BiVO_4_/N:NiFeO_x_ photoelectrodes possess higher absorption peak and longer carrier lifetime (2.69 μs) compared with BiVO_4_/NiFeO_x_ (1.77 μs) and BiVO_4_ (1.51 μs) photoanodes. Based on the above steady/transient spectra analysis, it can be concluded that N:NiFeO_x_ catalysts exhibited the preferable capability for promoting charge separation and extending the carriers lifetimes. Figure 3d shows their interfacial charge transfer (*η*_trans_) efficiencies for water oxidation reaction (Supplementary Fig. 18). The pristine BiVO_4_ exhibits a very low efficiency of 28% at 1.23 V_RHE_, while the surface deposition of NiFeO_x_ and N:NiFeO_x_ catalysts could effectively increase the *η*_trans_ efficiencies up to 66.3 and 88%, respectively. Furthermore, their electrochemical OER properties under dark conditions have also been studied and shown in Fig. 3e. Obviously, BiVO_4_/N:NiFeO_x_ possesses a lower overpotential and higher water oxidation current compared with BiVO_4_ and BiVO_4_/NiFeO_x_, further revealing its excellent OER activity. The large-scale fabrication of photoanodes should be necessarily required for future practical applications. Accordingly, the dual BiVO_4_/N:NiFeO_x_ photoanodes with a relatively large area (2 × 3.5 cm^2^) have been fabricated, and the photocurrent could achieve up to 37 mA at 1.23 V_RHE_ accompanied with an excellent stability of 10 h (Fig. 3f). Thus, the above results clearly demonstrate that the BiVO_4_/N:NiFeO_x_ photoanodes possess the tremendous potential for practical PEC water splitting applications.

### Effects of N-incorporation into BiVO_4_/NiFeO_x_ films

**Fig. 4:**
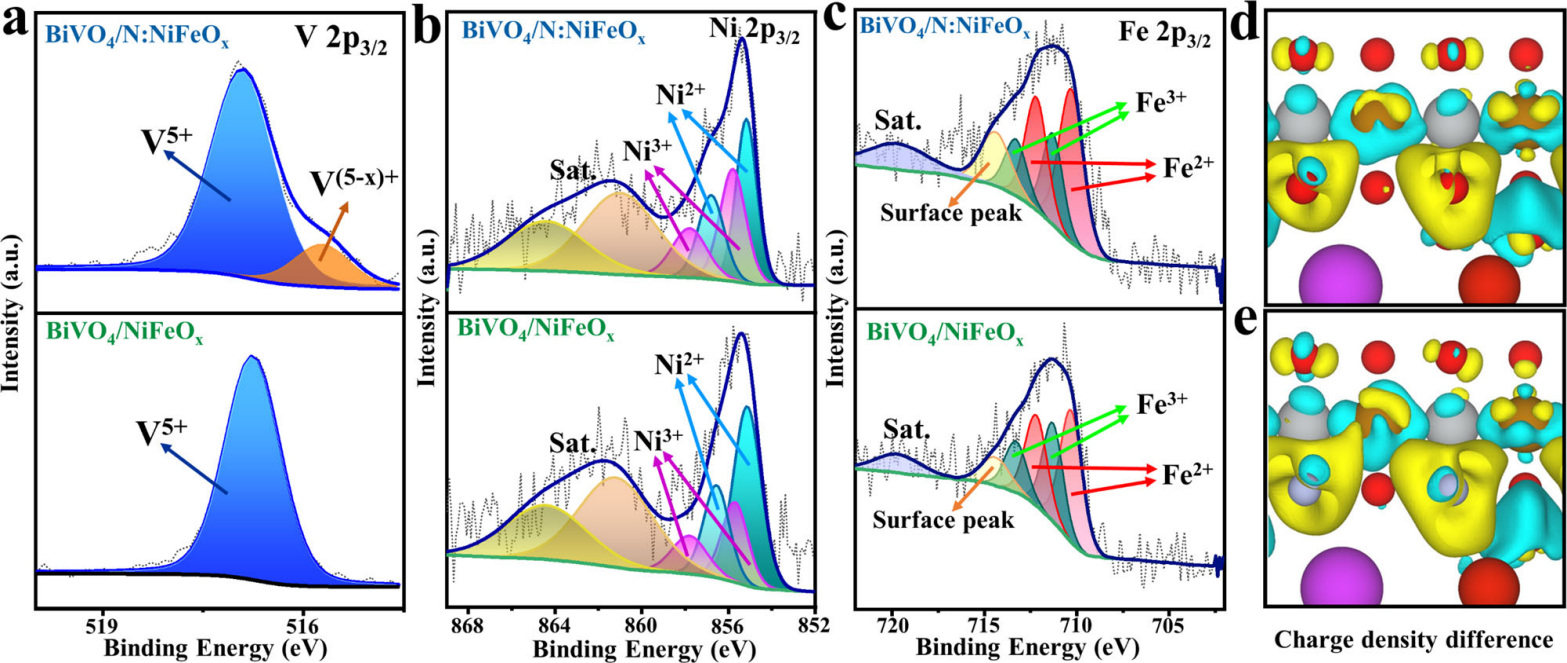
Effects of N-incorporation on the surface chemical states and electronic structures of BiVO_4_/NiFeO_x_. XPS high-resolution **a** V 2p, **b** Ni 2p, and **c** Fe 2p spectra for BiVO_4_/N:NiFeO_x_ and BiVO_4_/NiFeO_x_ photoanodes, respectively. Schematic of charge density difference (yellow and cyan represent charge accumulation and depletion, respectively; the cut-off of the density-difference isosurface is 0.01 Å^−3^) of BiVO_4_/NiFeO_x_ (**d**) and BiVO_4_/N:NiFeO_x_ (**e**).

Furthermore, the effects of the N-incorporation on the surface chemical states and electronic structures of both NiFeO_x_ and BiVO_4_ have been explored by XPS. As shown in Supplementary Figs. 19 and 20, no evident change could be detected in Bi 4f peaks of BiVO_4_/N:NiFeO_x_ compared with BiVO_4_/NiFeO_x_ and BiVO_4_, revealing the negligible influence of N-substitution on the Bi sites of BiVO_4_ photoanodes. Interestingly, compared with BiVO_4_/NiFeO_x_ samples, a shoulder peak at lower binding energy positions could be observed in V 2p spectra of BiVO_4_/N:NiFeO_x_ photoanodes (Fig. 4a), which should be attributed to the formation of V^(5−x)+^ species. Moreover, the relative ratio of Ni^3+^ species in BiVO_4_/N:NiFeO_x_ has been evidently increased (Fig. 4b)^46–50^, while the Fe^3+^ ratio has been decreased compared with BiVO_4_/NiFeO_x_ samples (Fig. 4c)^51–53^. On the basis of above results, it may be proposed that the partial substitution of O sites in NiFeO_x_ catalysts by N atoms should enrich the electron densities in Fe and Ni sites. Furthermore, the electron-enriched Ni sites conversely donated electrons to V sites of BiVO_4_ for restraining V^5+^ dissolution and improving the PEC stability. Additionally, the electron-enriched Fe sites could efficiently attract photo-generated holes from BiVO_4_ surfaces, which significantly promoted the oxygen-evolution activity. Thereby, the N-incorporation in NiFeO_x_ catalysts could effectively promote the oxygen evolution activity and stability of BiVO_4_ photoanodes. To further confirm the above speculations, the N atoms in N:NiFeO_x_ catalysts were replaced by O atoms again via an oxygen plasma treatment. As shown in Supplementary Figs. 20 and 21, all the XPS peaks of Fe, Ni, Bi, and V elements were nearly consistent with BiVO_4_/NiFeO_x_ photoanodes. Additionally, after the replacement of N with O atoms, the photocurrent density has been obviously reduced from 6.4 to 5 mA cm^−2^ at 1.23V_RHE_ accompanied by the poor PEC water oxidation stability (Supplementary Fig. 22), confirming the crucial roles of N-incorporation in promoting the OER activity and PEC stability of BiVO_4_ photoanodes.

### DFT calculation and analysis

Furthermore, the density functional theory (DFT) calculation has been performed to reveal the change of electron densities in Fe and Ni sites after incorporation of N atoms. As shown in Fig. 4d, e, the charge density difference results clearly reveal that the electron densities at Fe and Ni sites increased significantly (yellow regions) after partial substitution of O sites with N atoms in NiFeO_x_ catalysts. Additionally, the Bader charge analysis (Supplementary Table 4) also verified the enriched electron densities with the N-incorporation, which is highly consistent with the XPS results. Thereby, these calculation results could further provide supports on the crucial roles of N-incorporation in regulating the electronic structures of NiFeO_x_.

## Discussion

In summary, we reported a facile N-incorporation method to rationally regulate the electronic structures of NiFeO_x_ catalyst decorated on BiVO_4_ photoanodes. More detailed experiments and XPS analysis reveal that owing to the relatively low electronegativity of N atoms, their incorporation in NiFeO_x_ catalysts facilitates the electron enrichments in Fe and Ni sites. Furthermore, the Ni sites would donate electrons to V sites on BiVO_4_ surface, which could efficiently restrain V^5+^ dissolution and improve the PEC water oxidation stability. Moreover, the enhanced hole-attracting ability of Fe sites significantly promotes the oxygen-evolution activity. As expected, the BiVO_4_/N:NiFeO_x_ photoanodes exhibited an outstanding photocurrent density of 6.4 mA cm^−2^ at 1.23 V_RHE_ (AM 1.5 G, 100 mW cm^−2^) accompanying with the enhanced PEC stability. This work provides a new insight to construct highly efficient and stable OER catalysts for fabricating high-efficiency PEC devices.

## Methods

### Materials

All chemicals were of analytical grade purity, obtained from Sinopharm Chemical Reagent Co., Ltd., and used as received without further purification. Deionized water (Molecular Corp., 18.25 MΩ cm) used in the synthesis was from local sources.

### Synthesis of nanoporous BiVO_4_ photoanodes

The nanoporous BiVO_4_ photoanode was obtained based on the previous report^10^. 2 mM Bi(NO_3_)_3_·5H_2_O was dissolved in 0.4 M KI solution (50 mL). Then, the pH value of this solution was adjusted to 1.7 by HNO_3_. Subsequently, 0.23 M quinhydrone was dispersed into ethanol solution (20 mL). Finally, mixing the two solution and stirring vigorously for a few minutes to acquire the electrodeposited solution. The cathodic deposition was performed at a constant potential of −0.1 V vs. Ag/AgCl for 3 min at room temperature to obtain the BiOI electrodes, among which FTO, Ag/AgCl (4 M KCl), and platinum pair were used as working electrode (WE), reference electrode (RE) and counter electrode (CE), respectively. Then, VO(acac)_2_ (0.2 M, 0.2 mL) dissolved in DMSO (10 mL) solution was coated on the BiOI electrodes and heated in the air in a muffle furnace at 450 °C for 2 h (ramping rate = 2 °C/min) to convert to BiVO_4_. After calcination, the excess V_2_O_5_ on the electrode surface was soaked into NaOH (1 M) solution for 15 min to remove. Finally, the electrodes were rinsed with deionized water and dried in air to obtain pure BiVO_4_ photoanodes.

### Synthesis of BiVO_4_/NiFeO_x_ and BiVO_4_/N:NiFeO_x_ photoanodes

The as-prepared BiVO_4_ electrodes were immersed into the freshly mixed metal salt solution (pH~2.8) for 15 min (2.5 mL of 10 mM FeCl_3_•6H_2_O and 7.5 mL of 10 mM NiCl_2_•6H_2_O). Then, 2 M NaOH solution was added to adjust its pH to ~8 (The electrodes were still kept in this mixed solution during pH adjustment process). Subsequently, the solution was stood for 50 min and maintained at 25 °C throughout the co-catalyst loading process. Finally, the electrodes were washed by deionized water and calcined at 300 °C for 1 h in a muffle furnace in air atmosphere to obtain the BiVO_4_/NiFeO_x_ samples. The synthesis of BiVO_4_/N:NiFeO_x_ and BiVO_4_/O_2_-NiFeO_x_ photoanodes was the same as the above steps for the preparation of BiVO_4_/NiFeO_x_, except that the final calcination process is changed to a N_2_ or O_2_ plasma treatment for 5 min (a medium power of 10.5 W and a pressure of 300 Pa, Supplementary Fig. 23). The BiVO_4_/N:NiFeO_x_-O_2_ photoanodes were prepared via an oxygen plasma treatment BiVO_4_/N:NiFeO_x_ for 5 min.

### Synthesis of BiVO_4_/NiFeP and BiVO_4_/NiFeB photoanodes

The BiVO_4_ photoanodes were immersed into the fresh metal salt solution (2.5 mL of 10 mM FeCl_3_•6H_2_O and 7.5 mL of 10 mM NiCl_2_•6H_2_O), and then a NaBH_4_ aqueous solution was added dropwise. The solution was stood for 50 min. Finally, the electrodes were washed by deionized water to obtain the BiVO_4_/NiFeB photoanodes. Firstly, the BiVO_4_ films were dipped into a water solution (20 mL) A containing SnCl_2_ (0.8 g) and HCl (40 wt%, 0.8 mL) for 2 min. Secondly, the films were further immersed into a water solution (20 mL) B of PdCl_2_ (10 mM, 3.4 mL), HF (40–50 wt%, 0.16 mL) and HCl (40 wt%, 0.2 mL) for 2 min. Finally, the films were immersed into the solution C at 60 °C for 40 s and then rinsed with deionized water to obtain the BiVO_4_/NiFeP photoanodes. The water solution (20 mL) C contains NiSO_4_•6H_2_O (0.15 g), FeSO_4_•4H_2_O (0.15 g), NH_4_F (0.2 g), NaH_2_PO_2_•H_2_O (0.8 g), and Na_3_C_6_H_5_O_7_·2H_2_O (0.4 g), and the value of pH was further adjusted to 9.0 by adding ammonia^54^.

### Measurement and characterization

Scanning electron microscopy measurements were carried out on a field-emission scanning electron microscope (SEM, SU8020). Transmission electron microscopy (TEM) measurements were performed by using a FEI Tecnai TF20 microscope operated at 200 kV. The elemental composition and chemical valence states were explored by X-ray photoelectron spectroscopy (XPS, Al-Kα, 1486.6 eV, ESCALAB 250Xi). The crystalline structures were identified by X-ray diffraction analysis (XRD, Smartlab-SE). UV-visible diffuse reflectance spectra were performed on a UV-2550 (Shimadzu) spectrometer by using BaSO_4_ as the reference.

### Spectrum measurements

The photoluminescence (PL) spectra were tested on F-7000 fluorescence spectrophotometer (Hitachi, Tokyo Japan) under laser excitation of 355 nm. The time-resolved transient absorption (TA) spectra were performed on LP980 spectrometer (Edinburgh Instruments Ltd., model LP980), combined with a compact Q-switched Nd:YAG laser (Continuum, the USA). The probe source was a 150 W pulsed Xenon lamp for kinetic and spectral studies. The measurements were achieved with single-flash laser excitation at 355 nm (10 Hz, FWHM~7 ns) as the pump source. The kinetic traces and transient absorption spectra were collected with a Hamamatsu R928 photomultiplier tube detector (PMT) and an iStar ICCD camera (Andor Technology), respectively. The samples were placed in a film holder, which is suitable for semi-transparent materials. The obtained data were analyzed with the Edinburgh software (LP900). In addition, the decay curves were probed at 490 nm (Fig. 3c), and their fitting was based on a biexponential decay model according to the following equation and the fitting parameters have been listed in Supplementary Table 3.

1 $$\left({{{{{\rm{R}}}}}}({{{{{\rm{t}}}}}})={B}_{1}{e}^{(-\frac{t}{{\tau }_{1}})}+{B}_{2}{e}^{(-\frac{t}{{\tau }_{2}})}\right)$$

### Photoelectrochemical measurements

The photoelectrochemical measurement was carried out on an electrochemical workstation (CHI760E) in a standard three-electrode system and a 0.5 M K_3_BO_3_ electrolyte (pH = 9.5) under AM 1.5 G simulated sunlight (100 mW cm^−2^). A dual-channel power and energy meters (PM320E, THORLABS) equipped with high-sensitivity S310C probe (THORLABS) was used to calibrate the AM 1.5 light intensity to 100 mW/cm^2^. Moreover, the solar simulator used in our experiments has been equipped with a total-reflection mirror and AM 1.5 G fitter for PEC measurements and the corresponding spectrum has been measured by a spectrometer (BLUE-Wave, StellarNet) and shown in Supplementary Fig. 24. The photocurrent vs. voltage (*J*–*V*) characteristics were determined by scanning potential from −0.6 to 1.0 V (vs. Ag/AgCl) with a scan rate of 10 mV s ^−1^ and the applied potentials could be converted into reversible hydrogen electrode (RHE) using the Nernst equation:

2 $${E}_{{{{{{\mathrm{RHE}}}}}}}={E}_{{{{{{\mathrm{Ag}}}}}}/{{{{{\mathrm{AgCl}}}}}}}+0.059{{{{{\mathrm{pH}}}}}}+0.197(25\,^\circ {{{{{\rm{C}}}}}})$$

The incident photon to current efficiency (IPCE) was determined using a full solar simulator (Newport, Model 9600, 300 W Xe arc lamp) and a motorized monochromator (Oriel Cornerstone 130 1/8 m) at 0.6 V_RHE_ in a 0.5 M K_3_BO_3_ electrolyte. The IPCE result was calculated using the equation^55^:

3 $${{{{{\mathrm{IPCE}}}}}}( \% )=\frac{1240\times I({{{{{\mathrm{mA}}}}}}/{{{{{\mathrm{c}}}}}}{{{{{{\mathrm{m}}}}}}}^{2})}{{P}_{{{{{{\mathrm{light}}}}}}}({{{{{\mathrm{mW}}}}}}/{{{{{\mathrm{c}}}}}}{{{{{{\mathrm{m}}}}}}}^{2})\times \lambda ({{{{{\mathrm{nm}}}}}})}\times 100$$

where *I* is the measured photocurrent density at specific wavelength, λ is the wavelength of incident light, and *P*_light_ is the measured light power density at that wavelength.

Supposing 100% Faradaic efficiency, the half-cell applied bias photon-to-current efficiency (HC-ABPE) was calculated by following equation^55^:

4 $$({{{{{\mathrm{HC}}}}}}-{{{{{\mathrm{ABPE}}}}}})( \% )=\frac{I({{{{{\mathrm{mA}}}}}}/{{{{{\mathrm{c}}}}}}{{{{{{\mathrm{m}}}}}}}^{2})\times (1.23-{V}_{{{{{{\mathrm{bias}}}}}}})(V)}{{P}_{{{{{{\mathrm{light}}}}}}}({{{{{\mathrm{mW}}}}}}/{{{{{\mathrm{c}}}}}}{{{{{{\mathrm{m}}}}}}}^{2})}\times 100$$

where *I* is the photocurrent density, *V*_bias_ is the applied potential, and *P*_light_ is the incident illumination power density (100 mW cm^−2^).

The electrochemical impedance spectroscopy (EIS) Nyquist plots were obtained at 0.75 V (vs. RHE) with a small AC amplitude of 10 mV in the frequency range of 10^−2^ to 10^5^ Hz and the measured spectra were fitted with Zview software.

Surface charge transfer efficiencies (*η*_trans_) of BiVO_4_, BiVO_4_/NiFeO_x_, and BiVO_4_/N:NiFeO_x_ photoanodes can be calculated using the following equation^56^:

5 $${\eta }_{{{{{{\mathrm{trans}}}}}}}=\frac{{J}^{{{{{{{\mathrm{H}}}}}}}_{2}{{{{{\mathrm{O}}}}}}}}{{J}^{{{{{{{\mathrm{H}}}}}}}_{2}{{{{{{\mathrm{O}}}}}}}_{2}}}$$

${J}^{{{{{{{\mathrm{H}}}}}}}_{2}{{{{{\mathrm{O}}}}}}}$ and ${J}^{{{{{{{\mathrm{H}}}}}}}_{2}{{{{{{\mathrm{O}}}}}}}_{2}}$ are the photocurrent densities obtained in 0.5 M potassium borate electrolytes (pH 9.5) without and with H_2_O_2_, respectively. Additionally, a summary of recent significant progress of BiVO_4_-based photoanodes has been reviewed (Supplementary Table 5).

The evolution of H_2_ and O_2_ was performed in a 0.5 M K_3_BO_3_ electrolyte at 1.23 V_RHE_ under AM 1.5 G illumination (100 mW cm^−2^) by an online gas analysis system (Labsolar 6 A, Beijing Perfect light Technology Co. Ltd.) and a gas chromatograph (GC 7890 A, Agilent Technologies).

The PEC performances of two parallel BiVO_4_/N:NiFeO_x_ photoanodes (single area: 2 × 3.5 cm^2^, distance: ~1 cm) were performed at 1.23 V_RHE_ in 0.5 M K_3_BO_3_. Specifically, the simulated solar light illuminates vertically these two photoanodes, which were connected with copper wires.

### Computational method

The Vienna Ab Initio Simulation Package (VASP) code described by the projector augmented wave (PAW) method for ion-electron interaction was applied to the simulation calculations^57,58^. The generalized gradient approximation (GGA) expressed in the form of the Perdew-Burke-Ernzerhof (PBE) function was used to deal with exchange-correlation interactions^59^. The cutoff energy of 500 eV was taken into account by all calculations, and the Monkhorst-Pack k-point grid was set to 3 × 3 × 3 for bulk structure optimization, 5 × 5 × 1 for BiVO_4_(001)/NiFeO_x_ and BiVO_4_(001)/N:NiFeO_x_ heterostructures. The empirical correction scheme of Grimme (DFT+D2) was adopted for considering van der Waals (vdW) interaction^60^. The convergence criterion for Hellmann-Feynman forces and total energy were set to 0.01 eV/Å and 10^−5^ eV, and the vacuum space in the z-direction was greater than 20 Å to avoid the interaction between adjacent units during structural relaxation. A twelve atomic layers BiVO_4_ (001) slab model was used, and the bottom six atomic layers were fixed to simulate the bulk structure.

## Supplementary information

Supplementary Information

## Data Availability

Data reported in the main article are provided in the Source Data file. The remaining data that support the findings of this study are available from the corresponding author upon request. Source data are provided with this paper.

## Source data

Source Data
