# Supplementary material for: Nitrogen-incorporation activates NiFeOx catalysts for efficiently boosting oxygen evolution activity and stability of BiVO4 photoanodes
Source: Nat Commun. 2021 Nov 29;12:6969. doi: 10.1038/s41467-021-27299-0 (PMC8630083; doi:10.1038/s41467-021-27299-0)
Supplement: Supplementary file 1 — Supplementary Information [file 41467_2021_27299_MOESM1_ESM.pdf]

Supporting Information for

Nitrogen-Incorporation Activates $NiFeO_x$ Catalysts for Efficiently Boosting Oxygen Evolution Activity and Stability of $BiVO_4$ Photoanodes

Beibei Zhang,[1,2] Shiqiang Yu,[3] Ying Dai,[3] Xiaojuan Huang,[1] Lingjun Chou,*[1] Gongxuan Lu,*[1] Duojun Dong,[1]and Yingpu Bi*[1,4]

[1] State Key Laboratory for Oxo Synthesis & Selective Oxidation, National Engineering Research Center for Fine Petrochemical Intermediates, Lanzhou Institute of Chemical Physics, CAS, Lanzhou, 730000 (P. R. China)

[2] University of Chinese Academy of Sciences, Beijing 100049 (P. R. China).

[3] School of Physics, Shandong University, jinan 250100 (P. R. China).

[4] Dalian National Laboratory for Clean Energy, CAS, Dalian 116023 (P. R. China).

E-mail: ljchou@licp.cas.cn; gxlu@lzb.ac.cn; yingpubi@licp.cas.cn

Supplementary Chemical reagents

Bismuth nitrate pentahydrate ($Bi(NO_3)_3 \cdot 5H_2O$, 99%), potassium iodide (KI), quinhydrone, vanadium acetylacetone oxygen ($VO(acac)_2$), iron chloride hexahydrate ($FeCl_3 \cdot 6H_2O$, 99%), nickel chloride hexahydrate ($NiCl_2 \cdot 6H_2O$, 99%), dimethylsulfoxide (DMSO), ethyl alcohol, nitric acid (HNO_3), boric acid (H_3BO_3, 99%), potassium hydroxide (KOH, 99%) and sodium hydroxide (NaOH, 99%) were obtained from Sinopharm Chemical Reagent Co., Ltd. Deionized water with a resistivity of 18.25 MΩ·cm was used in all reactions. Fluorine-doped Tin Oxide (FTO) conductive glass was shaken by ultrasonic cleaning with acetone, ethanol, isopropyl alcohol and deionized water, respectively.

Supplementary Figures and Discussions

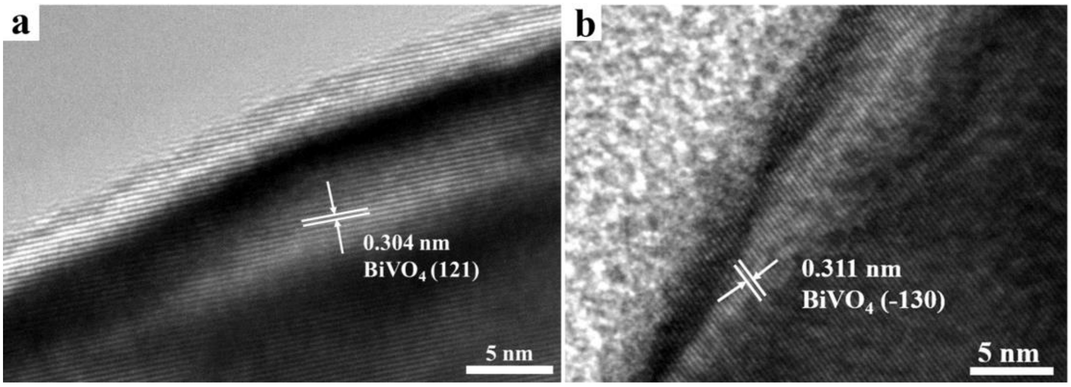

Supplementary Fig. 1 HR-TEM images (a, b) of the pristine $BiVO_4$ photoanodes.

Supplementary discussion

As shown in both SEM and TEM images, the $BiVO_4$ particles possess irregularly spherical structures, various crystal facets could be obtained during the HR-TEM measurements.

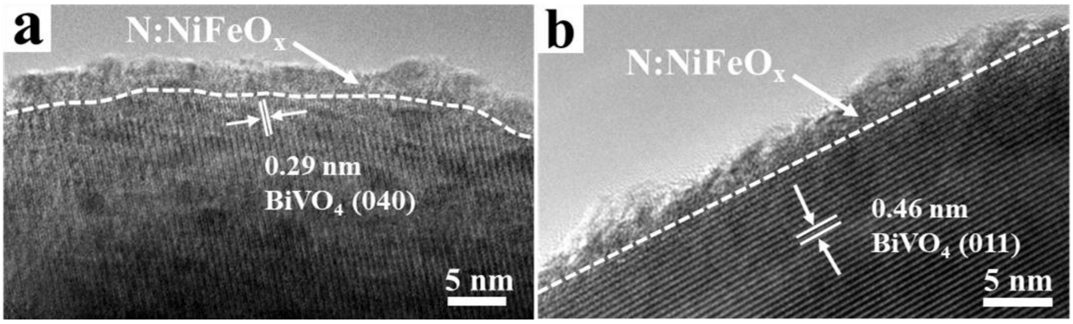

Supplementary Fig. 2 HR-TEM images of the $BiVO_4$/N:$NiFeO_x$ photoanodes.

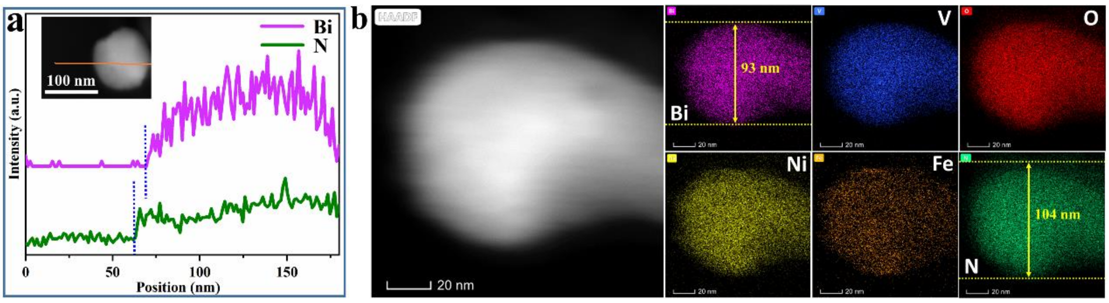

Supplementary Fig. 3 TEM-EDS line (a) and mapping (b) analysis for $BiVO_4$/N:$NiFeO_x$ photoanodes.

Supplementary discussion

The high-resolution line-scan analysis on $BiVO_4$/N:$NiFeO_x$ photoanodes has been performed. As shown in Supplementary Fig. 3a, before the appearance of Bi element, obvious N signal has been detected in the line-scan profiles. Additionally, the dimensions of Bi and N element distributions in the TEM-EDS mapping have been precisely measured (Gatan Digital Micrograph) and shown in Supplementary Fig. 3b, clearly confirming the larger distribution of N element (104 nm) than Bi element (93 nm). On the basis of above line-scan and mapping analysis, it was considered that the N element should mainly locate in the OER cocatalyst layer.

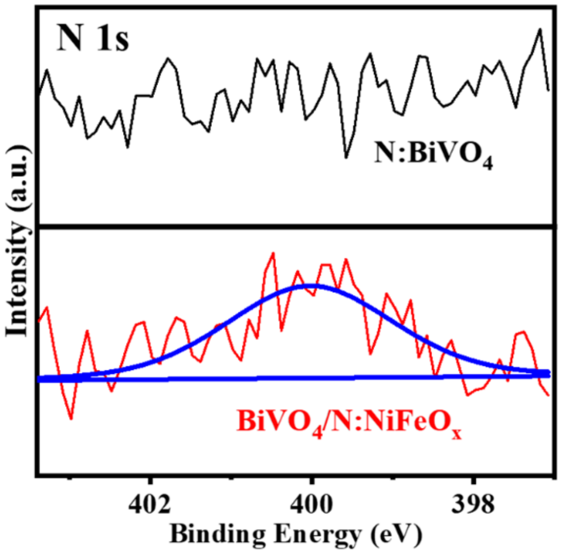

Supplementary Fig. 4 XPS high-resolution N 1s spectra for N:$BiVO_4$ and $BiVO_4$/N:$NiFeO_x$ photoanodes.

Supplementary discussion

For comparison, the pristine $BiVO_4$ photoanodes were also treated with N_2 plasma (marked as N:$BiVO_4$), while no evident N 1s peak could be detected from the XPS results (Supplementary Fig. 4). However, after N_2-plasma treatment on $BiVO_4$/$NiFeO_x$ photoanodes under the same conditions, there was an obvious N 1s peak located at 400 eV, suggesting that the nitrogen should be incorporated into the $NiFeO_x$ layer instead of $BiVO_4$. Additionally, according to the previously reported literatures,[1, 2] this N 1s peak at 397-402 eV should be attributed to the formation of N^{3-} states into the oxides by replacing O^{2-} sites.

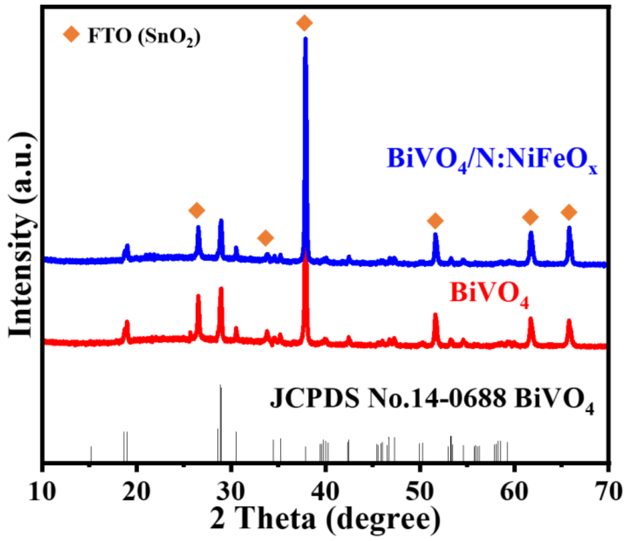

Supplementary Fig. 5 XRD patterns of $BiVO_4$ and $BiVO_4$/N:NiFeO$_x$ photoanodes.

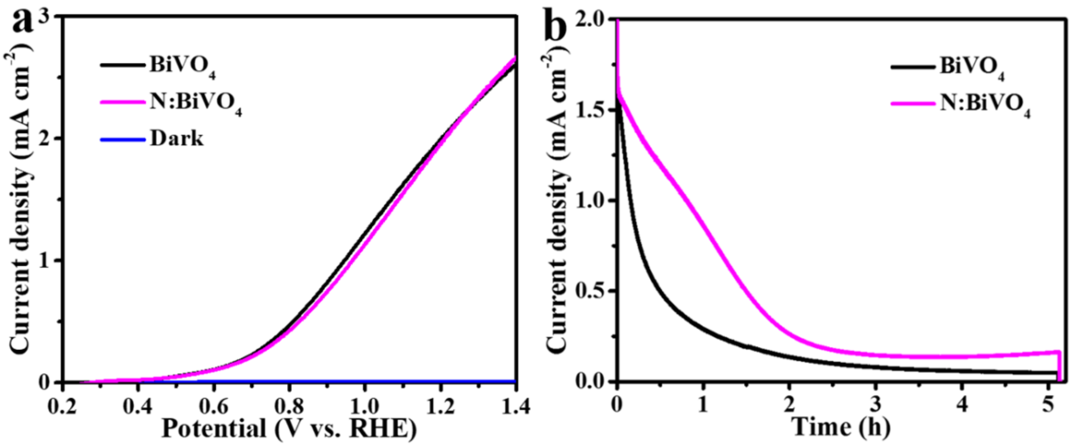

Supplementary Fig. 6 LSV (a) and i-t (b) curves for $BiVO_4$ photoanodes without and with N_2-plasma treatment.

Supplementary discussion

The pristine $BiVO_4$ photoanodes were also treated by the N_2-plasma for 5 minutes. As shown in Supplementary Fig. 6, compared with pristine $BiVO_4$, the photocurrent basically remained invariability and the stability was still very poor, which further indicates that the enhanced PEC performances of $BiVO_4$ should be mainly attributed to the N:NiFeO$_x$ catalysts by accelerating the OER process.

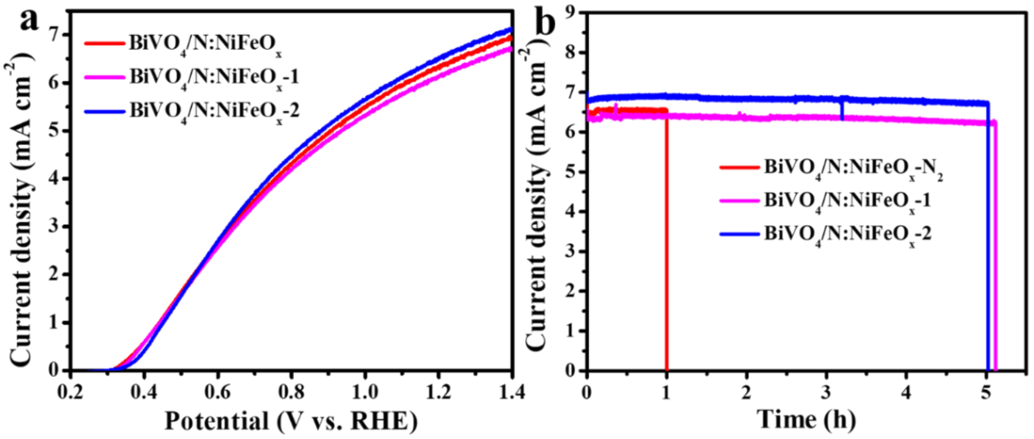

Supplementary Fig. 7 The repeatability of LSV (a) and i-t (b) curves for $BiVO_4$/N: $NiFeO_x$ photoanodes.

Supplementary discussion

Supplementary Fig. 7 shows the $BiVO_4$/N:$NiFeO_x$ photoanodes with excellent repeatability and stability. And the photocurrent density could reach up to 6.4±0.2 mA cm^{-2} at 1.23 V_{RHE} accompanied with outstanding stabilities.

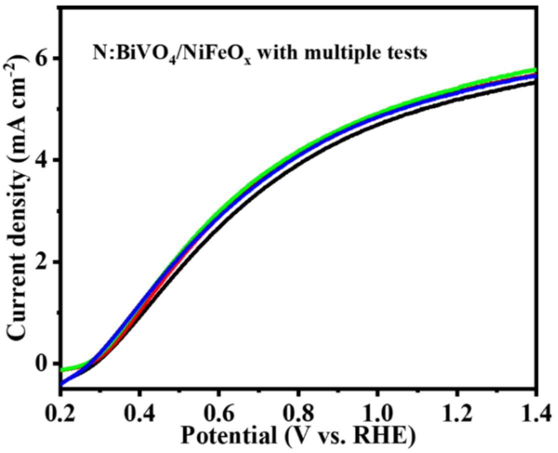

Supplementary Fig. 8 The multiple test LSV curves for N:$BiVO_4$/$NiFeO_x$ photoanodes measured at 0.5 M K_3BO_3 electrolyte.

Supplementary discussion

The PEC performances of N:$BiVO_4$/$NiFeO_x$ photoanodes have been evaluated and compared with $BiVO_4$/N:$NiFeO_x$. As shown in Supplementary Fig. 8, a photocurrent density of ~5.3 mA cm^{-2} (1.23 V_{RHE}) has been obtained on N:$BiVO_4$/$NiFeO_x$ photoanodes, which is much lower than that of $BiVO_4$/N:$NiFeO_x$ (6.4 mA cm^{-2}, 1.23 V_{RHE}). This demonstration further confirms that the N incorporation into $NiFeO_x$ layer could significantly promote the PEC activity of $BiVO_4$ photoanodes.

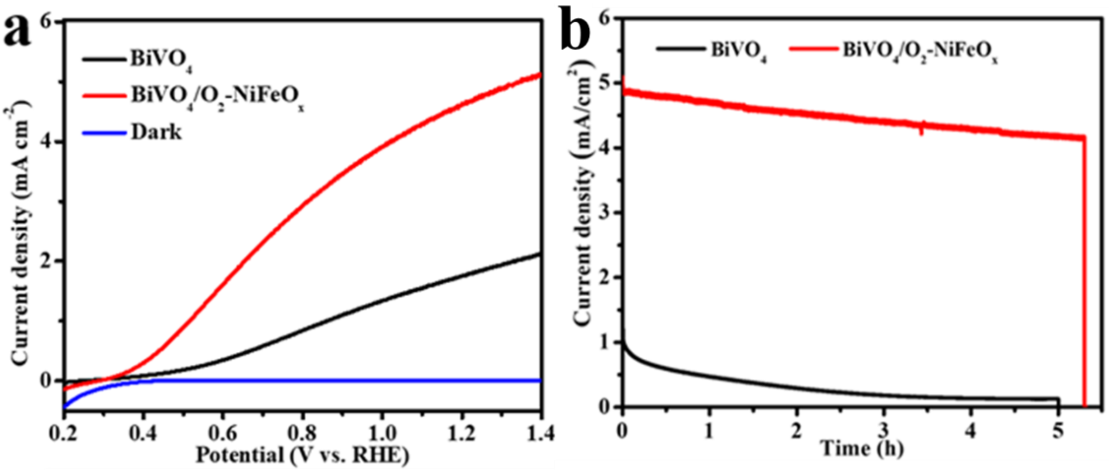

Supplementary Fig. 9 LSV curves (a) and I-t curves (b) of $BiVO_4$ and $BiVO_4/O_2$-$NiFeO_x$ photoanodes measured at 0.5 M K_3BO_3 electrolyte.

Supplementary discussion

To further clarify the effects of N atoms in N:$NiFeO_x$ catalysts on PEC activity and stability of $BiVO_4$ photoanodes, a facile O_2 plasma treatment process was performed on the $BiVO_4/NiFeO_x$ photoanodes. As shown in Supplementary Fig. 9. At 1.23 V_{RHE}, the $BiVO_4/O_2$-$NiFeO_x$ photoanode only exhibits a photocurrent density of 4.3 mA cm^{-2} accompanied by the poor PEC water oxidation stability, further demonstrating the crucial roles of N-incorporation in promoting the OER activity and PEC stability of $BiVO_4$ photoanodes.

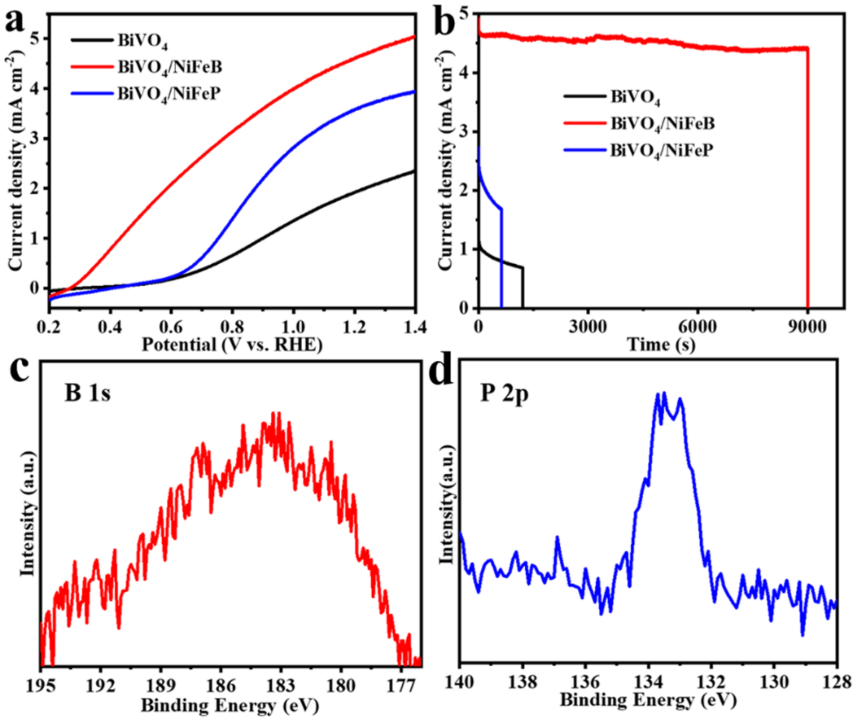

Supplementary Fig. 10 LSV (a) and i-t (b) curves for $BiVO_4$, $BiVO_4$/NiFeB and $BiVO_4$/NiFeP photoanodes; XPS high-resolution (c) B 1s and (d) P 2p spectra for $BiVO_4$/NiFeB and $BiVO_4$/NiFeP photoanodes, respectively.

Supplementary discussion

To further confirm the crucial roles of N-incorporation of $NiFeO_x$ for promoting the OER activity and PEC stability of $BiVO_4$ photoanodes, the PEC activities of NiFeB and NiFeP decorated $BiVO_4$ photoanodes have also been measured for comparisons. As shown in Supplementary Fig. 10a, both NiFeB and NiFeP catalysts could enhance the PEC water oxidation activities of $BiVO_4$ to a certain extent (4.7 mA cm^{-2} and 3.6 mA cm^{-2} at 1.23 V_{RHE}), which were still lower than that of $BiVO_4$/N:$NiFeO_x$ photoanodes (6.4 mA cm^{-2}). Besides, the PEC water oxidation stabilities of the two electrodes were gradually decreased with the prolonging of illumination time (Supplementary Fig. 10b). The comparative experiments demonstrate that compared with B and P atoms, the incorporation of N atoms with appropriate electronegativity could more effectively regulate the electronic structure of $NiFeO_x$ catalyst and promote the oxygen evolution activity and stability of $BiVO_4$ photoanodes.

Supplementary Fig. 10c and Fig. 10d show the B 1s and P 2p characteristic peaks of $BiVO_4$/NiFeB and $BiVO_4$/NiFeP photoanodes, indicating the successful incorporation of B and P atoms into the $NiFeO_x$ catalysts, respectively.

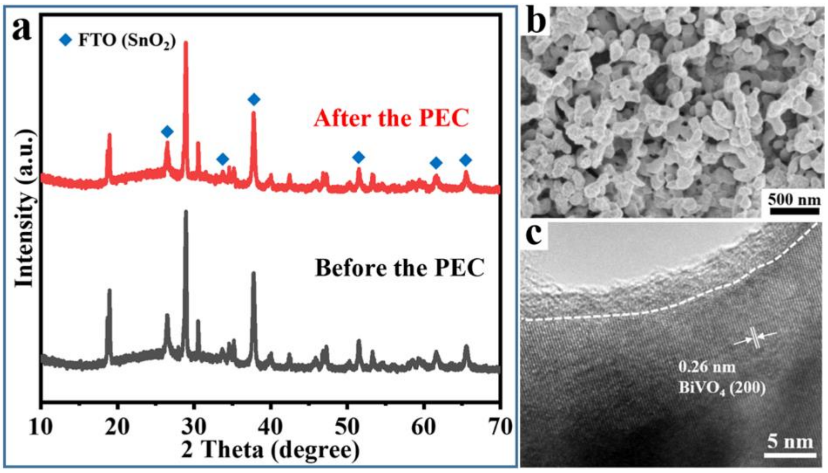

Supplementary Fig. 11 The XRD patterns (a), SEM image (b) and HR-TEM image (c) for $BiVO_4$/N:$NiFeO_x$ photoanodes after PEC measurements.

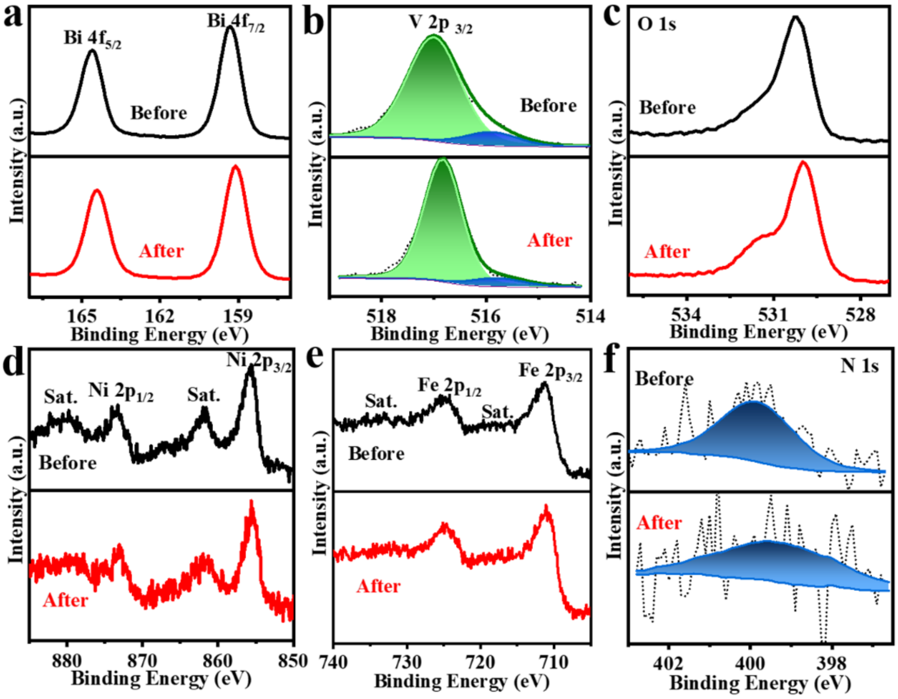

Supplementary Fig. 12 XPS high-resolution Bi 4f (a), V 2p (b), O 1s (c), Ni 2p (d), Fe 2p (e) and N 1s (f) spectra for $BiVO_4$/N:$NiFeO_x$ photoanodes before and after PEC measurements, respectively.

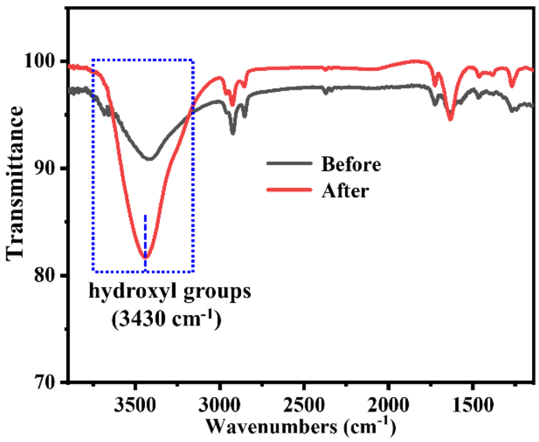

Supplementary Fig. 13 FTIR spectra for $BiVO_4$/N:$NiFeO_x$ samples before and after the PEC tests, respectively.

Supplementary discussion

It was considered that after the PEC stability tests, the increased noise of N1s spectrum (Supplementary Fig. 12f) should be mainly attributed to the formation of hydroxyl (OH) groups on the photoanode surfaces. More specifically, compared with the fresh samples, the detection sensitivity on the photoanode surface has been relatively decreased, especially for the light and low-content elements. To further confirm the above speculation, Fourier transform infrared (FTIR) spectroscopy has been employed to explore the changes of OH groups on the photoanode surface (Supplementary Fig. 13). Obviously, after the PEC stability tests, the stretching vibration peak of OH groups at 3430 cm^{-1} has been significantly increased, which clearly reveal the OH formation on the photoanode surface.

Supplementary Table 1 The elemental composition analysis from XPS for $BiVO_4$/N:$NiFeO_x$ photoanodes before and after PEC measurements.

Element	Atomic % (before PEC test)	The proportion of relative to C 1s (before PEC test)	Atomic % (after PEC test)	The proportion of relative to C 1s (after PEC test)
Bi 4f	7.1	17.07%	9.89	21.18%
C 1s	41.6	100%	46.69	100%
Fe 2p	5	12.02%	4.05	8.67%
N 1s	1.94	4.66%	1.48	3.17%
Ni 2p	2.43	5.84%	1.68	3.6%
O 1s	36.55	87.86%	33.32	71.36%
V 2p	5.38	12.93%	2.89	6.19%

Supplementary discussion

The SEM, TEM, XRD and XPS results for the $BiVO_4$/N:$NiFeO_x$ photoanodes after the PEC tests have been performed. As shown in Supplementary Fig. 11, after the stability test, the morphology and crystalline structures of $BiVO_4$/N:$NiFeO_x$ have no evident changes compared with the fresh samples (Fig. 1). Additionally, the HR-TEM images (Supplementary Fig. 11c) also clearly indicate that the N:$NiFeO_x$ cocatalyst layers were still uniformly covered on $BiVO_4$ surfaces with the thickness of ~4 nm, indicating it's excellent structural stability. Moreover, as shown in Supplementary Fig. 12 and Table 1, the XPS results reveal that the characteristic peaks of all elements could still be detected after the PEC stability tests, but their contents have been slightly decreased compared with the pristine samples, especially for V element, which should be due to the photo-induced V^{5+} dissolution from the $BiVO_4$ lattices.[3-5]

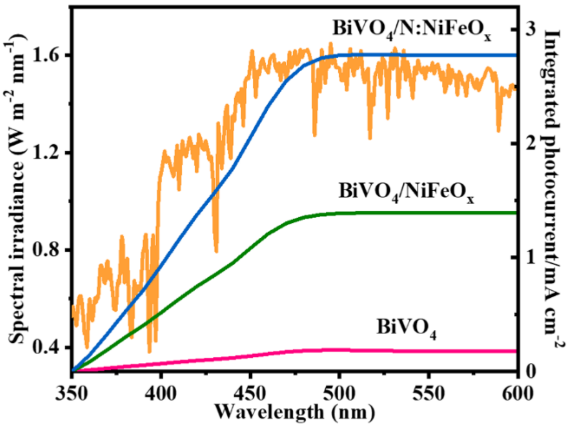

Supplementary Fig. 14 Calculated photocurrent density curves by integrating IPCE curves in (Fig. 2d) with the standard solar spectrum.

Supplementary discussion

The estimated photocurrent densities (J_c) were calculated by integrating the IPCE values with the standard solar spectrum (ASTMG-173-03) using the following equation:

$$J_c = \int_{350}^{600} \frac{\lambda \times IPCE(\lambda) \times E(\lambda)}{1240} d(\lambda) \quad (1)$$

Specifically, λ and E(λ) represent the light wavelength (nm) and the corresponding power density (mW cm^{-2}), respectively. According to the above equation, the calculated photocurrent densities for $BiVO_4$, $BiVO_4/NiFeO_x$ and $BiVO_4/N:NiFeO_x$ photoanodes were 0.17, 1.39 and 2.77 mA cm^{-2} at 0.6 V_{RHE}, respectively, which are all close to the measured values (0.13, 1.14 and 2.65 mA cm^{-2} for $BiVO_4$, $BiVO_4/NiFeO_x$ and $BiVO_4/N:NiFeO_x$, Fig. 2a). These demonstrations clearly reveal that the simulated AM 1.5G solar light was well matched with the standard solar spectrum.

Supplementary Table 2 The fitted results of EIS data using the equivalent circuit in Fig. 2e

Samples	R_{ct}/Ω	CPE/(F cm^{-2})
$BiVO_4$	458.9	1.47×10^{-4}
$BiVO_4$/$NiFeO_x$	149.8	1.40×10^{-4}
$BiVO_4$/N:$NiFeO_x$	139.5	1.80×10^{-4}

Supplementary discussion

The values of CPE have been evaluated and shown in Supplementary Table 2. It can be seen that the $BiVO_4$/N:$NiFeO_x$ photoanode shows a higher value of CPE than both $BiVO_4$ and $BiVO_4$/$NiFeO_x$, indicating its more efficient charge transfer from the electrode/electrolyte interfaces.[6, 7]

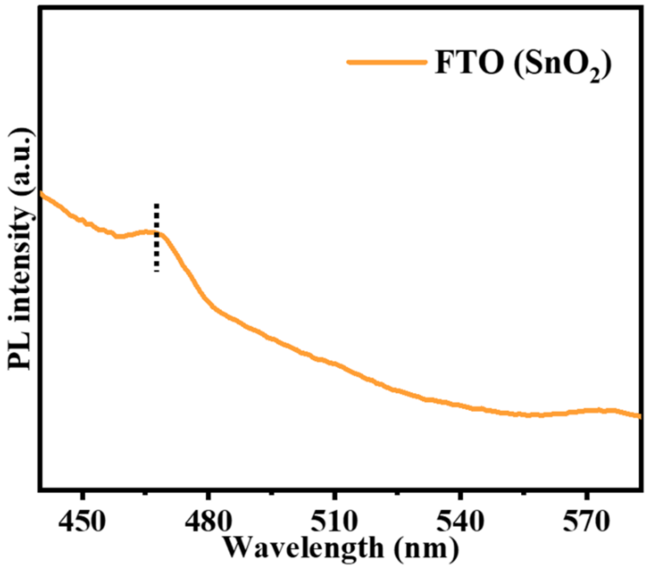

Supplementary Fig. 15 PL spectrum of FTO (SnO_2) by F-7000 fluorescence spectrophotometer under laser excitation of 355 nm.

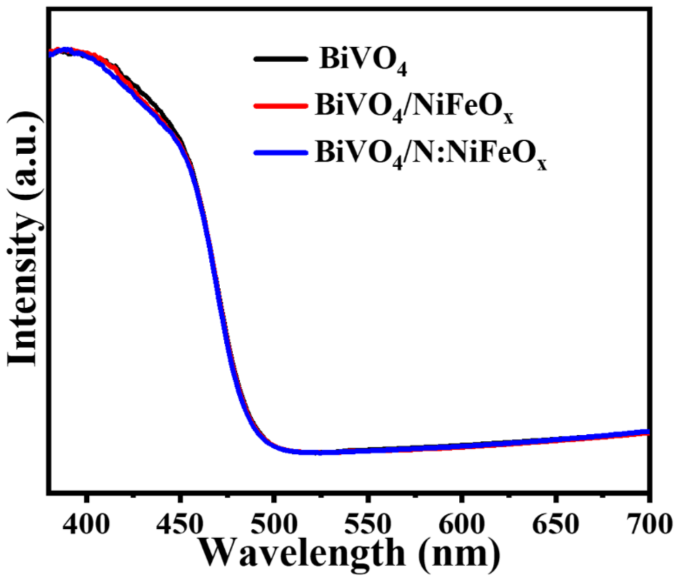

Supplementary Fig. 16 UV-vis diffuse reflectance spectra of $BiVO_4$, $BiVO_4/NiFeO_x$ and $BiVO_4/N:NiFeO_x$ photoanodes.

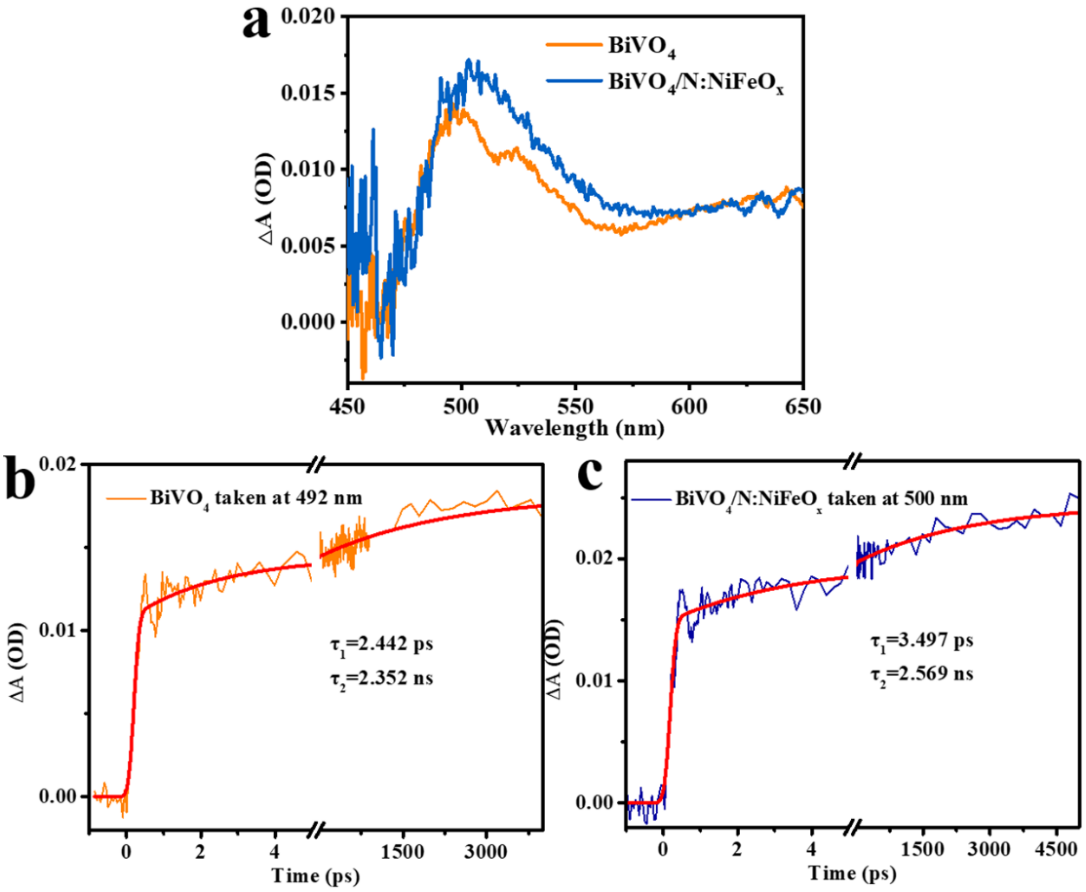

Supplementary Fig. 17 The ultrafast transient absorption spectroscopy (fs-TAS) of $BiVO_4$ and $BiVO_4/N:NiFeO_x$ photoanodes (a), TAS decay kinetic traces characteristic to $BiVO_4$ (b, taken at 492 nm) and $BiVO_4/N:NiFeO_x$ (c, taken at 500 nm) photoanodes.

Supplementary discussion

The ultrafast transient absorption spectroscopy (fs-TAS) has been performed and shown in Supplementary Fig. 17. It can be clearly observed that compared with pristine $BiVO_4$ photoanodes, the incorporation of N:$NiFeO_x$ cocatalysts could significantly increase the hole carriers lifetime from ~2.352 to ~2.569 ns.[8-11] The fs-TAS results demonstrated that N:$NiFeO_x$ cocatalysts could effectively promote charge separation and extend the carriers lifetimes, which is consistent with the ns-TAS results shown in Fig. 3c.

Supplementary Table 3 Fitting parameters of the TA kinetic tracked at 490 nm

Samples	A	τ
$BiVO_4$	2.43	1.51 μs
$BiVO_4$/$NiFeO_x$	1.82	1.77 μs
$BiVO_4$/N:$NiFeO_x$	0.95	2.69 μs

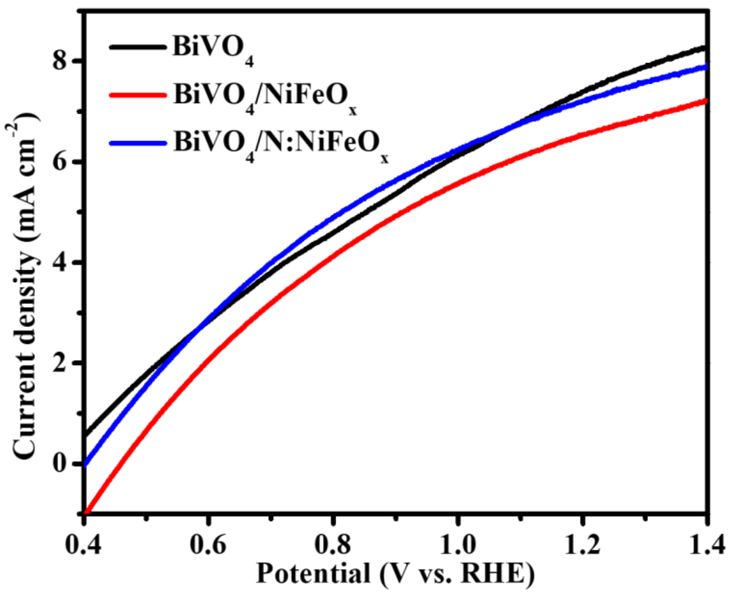

Supplementary Fig. 18 LSV curves of $BiVO_4$, $BiVO_4/NiFeO_x$ and $BiVO_4/N:NiFeO_x$ photoanodes measured at 0.5 M K_3BO_3 electrolyte with H_2O_2 as the sacrifice reagent.

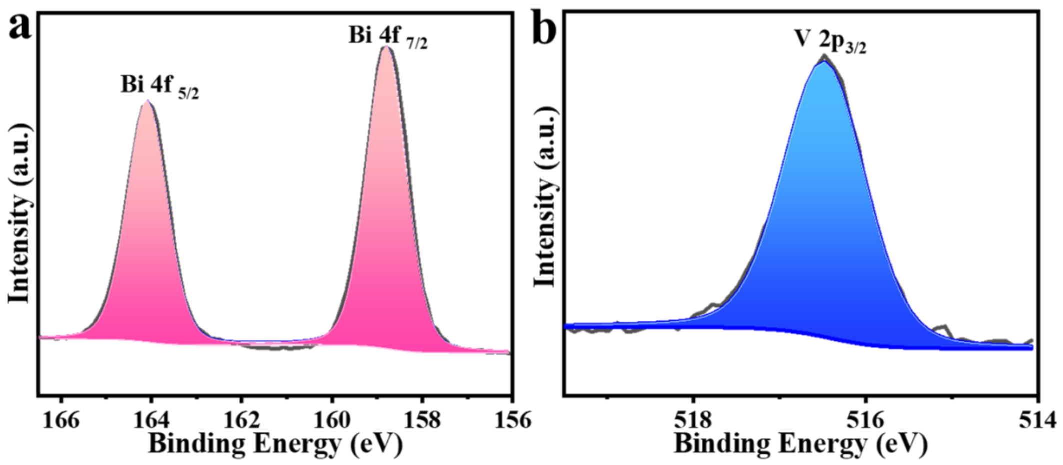

Supplementary Fig. 19 XPS high-resolution (a) Bi 4f and (b) V 2p spectra for $BiVO_4$ photoanodes.

Supplementary discussion

Supplementary Fig. 19 shows the characteristic peaks of Bi 4f and V 2p of pristine $BiVO_4$, which are consistent with the literature reports.[12]

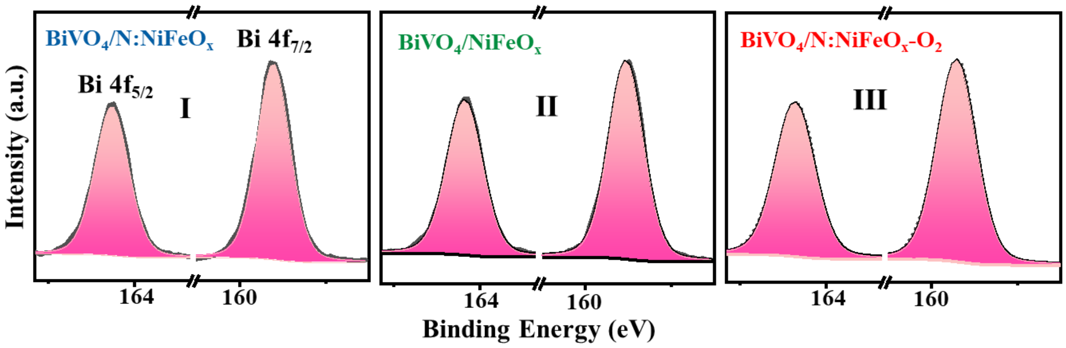

Supplementary Fig. 20 XPS high-resolution Bi 4f spectra for $BiVO_4$/N:$NiFeO_x$, $BiVO_4$/$NiFeO_x$ and $BiVO_4$/N:$NiFeO_x$-O_2 photoanodes, respectively.

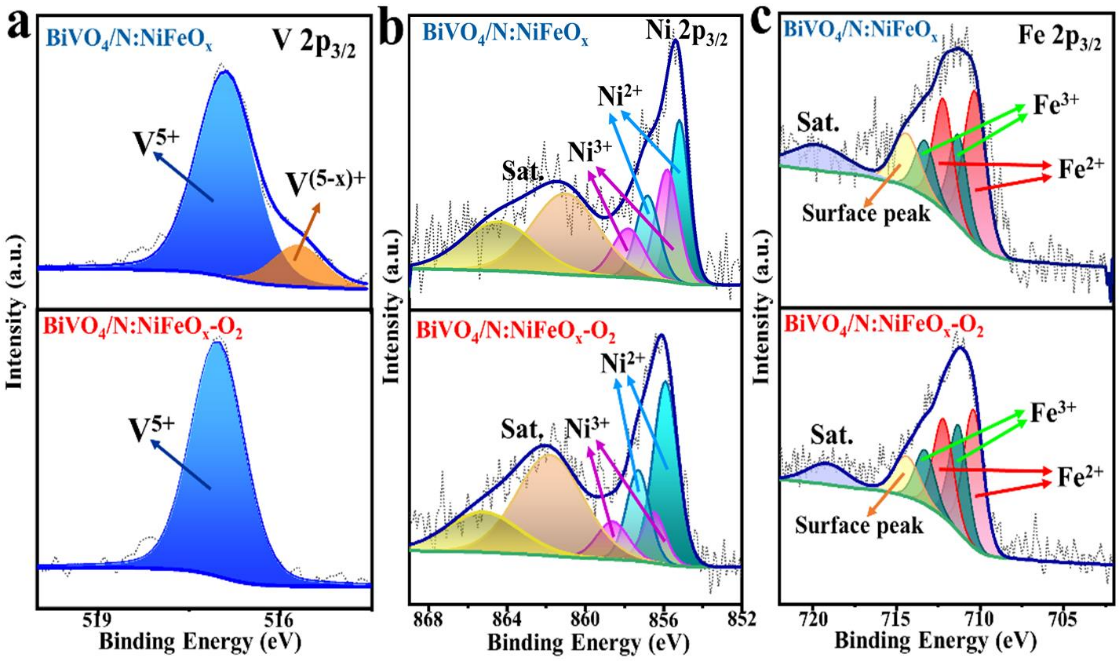

Supplementary Fig. 21 XPS high-resolution (a) V 2p, (b) Ni 2p and (c) Fe 2p spectra for $BiVO_4$/N:$NiFeO_x$ and $BiVO_4$/N:$NiFeO_x$-O_2 photoanodes.

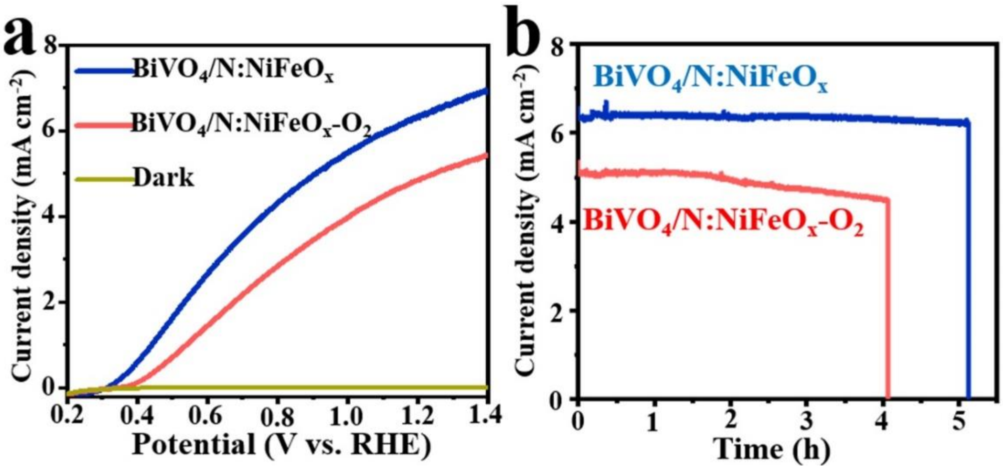

Supplementary Fig. 22 (a) LSV and (b) i-t curves of $BiVO_4$/N:NiFeO_x and $BiVO_4$/N:NiFeO_x-O_2 photoanodes.

Supplementary Table 4 Bader charges of V, Bi, Fe and Ni sites.

Site	Bader charges (e) FeNiO_x	Bader charges (e) N:FeNiO_x
**V27**	3.15	3.17
**Bi36**	3.08	3.086
**Fe1**	6.66	6.71
**Fe2**	6.62	6.67
**Ni1**	8.82	8.84
**Ni2**	8.77	8.80

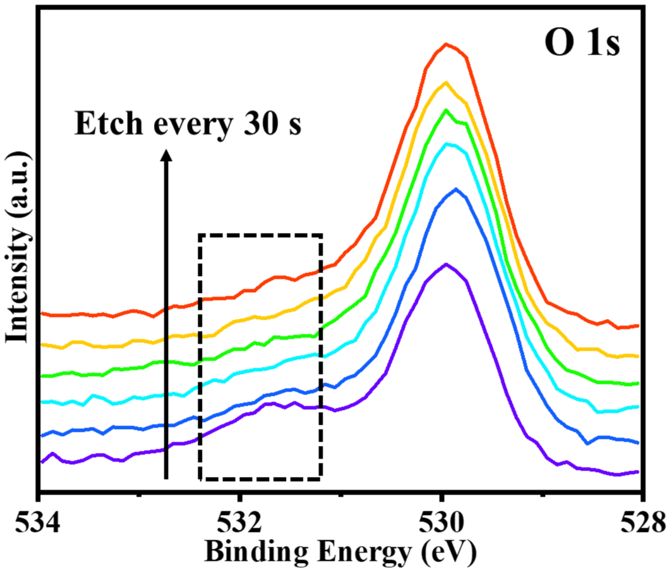

Supplementary Fig. 23 XPS high-resolution O1s spectra for $BiVO_4/NiFeO_x$ photoanodes.

Supplementary discussion

Supplementary Fig. 23 shows the variation of O 1s peaks with the etching time. It can be seen that the peak of hydroxyl disappears gradually, indicating that the sample after N_2-plasma etching treatment exists in the form of iron and nickel oxides.

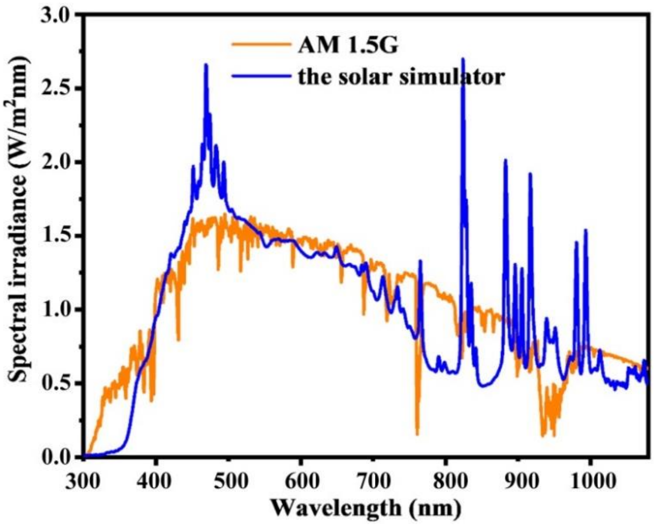

Supplementary Fig. 24 The spectrum comparison of our solar simulator with the standard AM 1.5G.

Supplementary discussion

The solar simulator used in our experiments has been equipped with a total-reflection mirror and AM 1.5G fitter for PEC measurements. The spectrum of our solar simulator has been measured by a spectrometer (BLUE-Wave, StellarNet), which is basically matched with the AM 1.5G standard spectrum (Supplementary Fig. 24).

Supplementary Table 5 Summary of recent significant progress of $BiVO_4$-based photoanodes

**Photoanodes**	**Photocurrent(1.23V_{RHE})**	**Published year**	**Ref.**
$BiVO_4$/FeOOH/NiOOH	4.5 mA/cm^2	2014	13
WO_3/(W, Mo)-$BiVO_4$	5.35 mA/cm^2	2014	14
N_2-$BiVO_4$/FeOOH/NiOOH	5.1 mA/cm^2	2015	12
WO_3/$BiVO_4$/CoPi	6.72 mA/cm^2	2015	15
Mo:$BiVO_4$/Fe(Ni)OOH	5.82 mA/cm^2	2016	16
Ni:FeOOH/WO_3/$BiVO_4$	4.5 mA/cm^2	2016	17
NiOOH/FeOOH/CQD/$BiVO_4$	5.99 mA/cm^2	2017	18
$BiVO_4$/FeOOH/NiOOH	4.8 mA/cm^2	2018	3
$FeCoO_x$/$BiVO_4$	4.82 mA/cm^2	2018	19
Mo:$BiVO_4$/TANF	5.1 mA/cm^2	2018	20
Dual $BiVO_4$/FeOOH/NiOOH	5.87 mA/cm^2	2018	21
$BiVO_4$/TiO_{2-x}	6.12 mA/cm^2	2019	22
NiOOH/BP/$BiVO_4$	4.48 mA/cm^2	2019	23
LBSO/Mo:$BiVO_4$	[a]5.15 mA/cm^2	2019	24
	[b]6.22 mA/cm^2		
Co@CB[5]/$BiVO_4$	4. 8 mA/cm^2	2020	25
Ov-$BiVO_4$@NiFe-MOFs	5.3 mA/cm^2	2021	26
$NiFeO_x$/$BiVO_4$	[a]5.54 mA/cm^2	2020	27
	[b]6.24 mA/cm^2		
$BiVO_4$/N:$NiFeO_x$	6.4 mA/cm^2	This work	

[a)] Photocurrents obtained by using single-photoanode configuration.

[b)] Photocurrents obtained by using dual-photoanode configuration.

Supplementary References

[1] Yang, X., Wolcott, A., Wang, G., Sobo, A., Fitzmorris, R. C., Qian, F., Zhang, J. Z. & Li, Y. Nitrogen-Doped ZnO Nanowire Arrays for Photoelectrochemical Water Splitting. *Nano Lett.* **9**, 2331-2336 (2009).

[2] Wang, J., Tafen, D. N., Lewis, J. P., Hong, Z., Manivannan, A., Zhi, M., Li, M. & Wu, N. Origin of Photocatalytic Activity of Nitrogen-Doped TiO_2 Nanobelts. *J. Am. Chem. Soc.* **131**, 12290-12297 (2009).

[3] Lee, D. K. & Choi, K. S. Enhancing long-term photostability of $BiVO_4$ photoanodes for solar water splitting by tuning electrolyte composition. *Nat. Energy* **3**, 53-60 (2018).

[4] Kuang, Y. et. al. Ultrastable low-bias water splitting photoanodes via photocorrosion inhibition and in situ catalyst regeneration. *Nat. Energy,* **2**, 16191 (2017).

[5] Berglund, S. P., Flaherty, D. W., Hahn, N.T., Bard, A. J. & Mullins, C. B. Photoelectrochemical Oxidation of Water Using Nanostructured $BiVO_4$ Films. *J. Phys. Chem. C* **115**, 3794-3802 (2011).

[6] Malara, F. et. al. Direct Observation of Photoinduced Higher Oxidation States at a Semiconductor/Electrocatalyst Junction. *ACS Catal*. **10**, 10476-10487 (2020).

[7] Klahr, B., Gimenez, S., Fabregat-Santiago, F., Hamann, T. & Bisquert, J. Water Oxidation at Hematite Photoelectrodes: The Role of Surface States. *J. Am. Chem. Soc.* **134**, 4294-4302 (2012).

[8] Kahraman, A., Vishlaghi, M. B., Baylam, I., Sennaroglu, A. & Kaya, S. Roles of Charge Carriers in the Excited State Dynamics of $BiVO_4$ Photoanodes. *J. Phys. Chem. C* **123**, 28576-28583 (2019).

[9] Ravensbergen, J., Abdi, F. F., van Santen, J. H., Frese, R. N., Dam, B. van de Krol, R. & Kennis, J. T. M. Unraveling the Carrier Dynamics of $BiVO_4$: A Femtosecond to Microsecond Transient Absorption Study. *J. Phys. Chem. C* **118**, 27793-27800 (2014).

[10] Ma, Y., Pendlebury, S. R., Reynal, A., Le Formal, F. & Durrant, J. R. Dynamics of photogenerated holes in undoped $BiVO_4$ photoanodes for solar water oxidation. *Chem. Sci*. **5**, 2964-2973 (2014).

[11] Aiga, N., Jia, Q., Watanabe, K., Kudo, A., Sugimoto, T. & Matsumoto, Y. Electron-Phonon Coupling Dynamics at Oxygen Evolution Sites of Visible-Light-Driven Photocatalyst: Bismuth Vanadate. *J. Phys. Chem. C* **117**, 9881-9886 (2013).

[12] Kim, T. W., Ping, Y., Galli, G. A. & Choi, K. S. Simultaneous enhancements in photon absorption and charge transport of bismuth vanadate photoanodes for solar water splitting. *Nat. Commun.* **6**, 8769 (2015).

[13] Kim, T. W. & Choi, K.-S. Nanoporous $BiVO_4$ Photoanodes with Dual-Layer Oxygen Evolution Catalysts for Solar Water Splitting. *Science* **343**, 990-994 (2014).

[14] Shi, X., Choi, I. Y., Zhang, K., Kwon, J., Kim, D. Y., Lee, J. K., Oh, S. H., Kim, J. K. & Park, J. H. Efficient photoelectrochemical hydrogen production from bismuth vanadate-decorated tungsten trioxide helix nanostructures. *Nat Commun.* **5**, 4775 (2014).

[15] Pihosh, Y. et. al. Photocatalytic generation of hydrogen by core-shell $WO_3/BiVO_4$ nanorods with ultimate water splitting efficiency. *Sci. Rep.* **5**, 11141 (2015).

[16] Qiu, Y. et. al. Efficient solar-driven water splitting by nanocone $BiVO_4$-perovskite tandem cells. *Sci. Adv.* **2**, e1501764 (2016).

[17] Cai, L., Zhao, J., Li, H., Park, J., Cho, I. S., Han, H. S. & Zheng, X. One-Step Hydrothermal Deposition of Ni:FeOOH onto Photoanodes for Enhanced Water Oxidation. *ACS Energy Lett.* **1**, 624-632 (2016).

[18] Ye, K.-H., Wang, Z., Gu, J., Xiao, S., Yuan, Y., Zhu, Y., Zhang, Y., Mai, W. & Yang, S. Carbon

quantum dots as a visible light sensitizer to significantly increase the solar water splitting performance of bismuth vanadate photoanodes. *Energy Environ. Sci.* **10**, 772-779 (2017).

[19] Wang, S., He, T., Yun, J.-H., Hu, Y., Xiao, M., Du, A. & Wang, L. New Iron-Cobalt Oxide Catalysts Promoting $BiVO_4$ Films for Photoelectrochemical Water Splitting. *Adv. Funct. Mater.* **28**. 1802685 (2018).

[20] Shi, Y., Yu, Y., Yu, Y., Huang, Y., Zhao, B. & Zhang, B. Boosting Photoelectrochemical Water Oxidation Activity and Stability of Mo-Doped $BiVO_4$ through the Uniform Assembly Coating of NiFe-Phenolic Networks. *ACS Energy Lett.* **3**, 1648-1654 (2018).

[21] Wang, S., Chen, P., Bai, Y., Yun, J. H., Liu, G. & Wang, L. New $BiVO_4$ Dual Photoanodes with Enriched Oxygen Vacancies for Efficient Solar-Driven Water Splitting. *Adv. Mater.* **30**, 1800486 (2018).

[22] Tian, Z., Zhang, P., Qin, P., Sun, D., Zhang, S., Guo, X., Zhao, W., Zhao, D. & Huang, F. Novel Black $BiVO_4/TiO_{2-x}$ Photoanode with Enhanced Photon Absorption and Charge Separation for Efficient and Stable Solar Water Splitting. *Adv. Energy Mater.* **9**, 1901287 (2019).

[23] Zhang, K., Jin, B., Park, C., Cho, Y., Song, X., Shi, X., Zhang, S., Kim, W., Zeng, H. & Park, J. H. Black phosphorene as a hole extraction layer boosting solar water splitting of oxygen evolution catalysts. *Nat. Commun.* **10**, 2001 (2019).

[24] Jian, J. et. al. Embedding laser generated nanocrystals in $BiVO_4$ photoanode for efficient photoelectrochemical water splitting. *Nat. Commun.* **10**, 2609 (2019).

[25] Li, F., Yang, H., Zhuo, Q., Zhou, D., Wu, X., Zhang, P., Yao, Z. & Sun, L. A Cobalt@Cucurbit[5]uril Complex as a Highly Efficient Supramolecular Catalyst for Electrochemical and Photoelectrochemical Water Splitting. *Angew. Chem. Int. Ed*. **60**, 1976-1985 (2021).

[26] Pan, J. et. al. Activity and Stability Boosting of an Oxygen-Vacancy-Rich $BiVO_4$ Photoanode by NiFe-MOFs Thin Layer for Water Oxidation. *Angew. Chem. Int. Ed*. **60**, 1433-1440 (2021).

[27] Wang, S., He, T., Chen, P., Du, A., Ostrikov, K. K., Huang, W. & Wang, L. In Situ Formation of Oxygen Vacancies Achieving Near-Complete Charge Separation in Planar $BiVO_4$ Photoanodes. *Adv. Mater.* **32**, 2001385 (2020).
